# Integrating the “best” evidence into nursing of venous thromboembolism in ICU patients using the i-PARIHS framework

**DOI:** 10.1371/journal.pone.0237342

**Published:** 2020-08-06

**Authors:** Xia Qin, Pin Yu, Huiling Li, Yan Hu, Xuehua Li, Qin Wang, Lu Lin, Li Tian

**Affiliations:** 1 The First People’s Hospital of Kunshan, Suzhou, China; 2 The First Affiliated Hospital of Soochow University, Suzhou, China; 3 School of Nursing, Medical College of Soochow University, Suzhou, China; 4 School of Nursing, Fudan University, Shanghai, China; University of Adelaide, AUSTRALIA

## Abstract

**Objectives:**

To explore how to integrate the “best” practice into nursing of venous thromboembolism (VTE) based on the integrated-Promoting Action on Research Implementation in Health Services (i-PARIHS) framework.

**Methods:**

A mixed-methods design was used. A steering group for clinical evidence implementation (EI) was established to conduct pre-implementation baseline surveys, a thorough analysis of the evidence, and an analysis of the survey results. The hindering and enabling factors associated with the clinical implementation of the evidence were analysed based on the three core elements of i-PARIHS, to formulate the clinical implementation plan for VTE nursing evidence. On-site expert reviews and focus group interviews were used to evaluate the feasibility of the draft plan, make adjustments, and finalize the evidence-based practice plan, which was then put into practice and evaluated.

**Results:**

A new nursing process, a health education manual and a nursing quality checklist on VTE has been established and proved to be appropriate through the implementation. Compliance with evidence related to VTE nursing increased significantly in the two units, with better compliance in unit B than unit A. The knowledge, attitude and behaviour scores for VTE nursing increased substantially in both nurses and patients.

**Conclusion:**

The EI programme of incorporating the “best” evidence on VTE nursing into clinical practice using the i-PARIHS framework demonstrated feasibility, appropriateness and effectiveness and could serve as a reference.

## Background

Venous thromboembolism (VTE), encompassing deep venous thrombosis (DVT) and pulmonary embolus (PE), has been deemed a major threat to the safety of hospitalized patients [1]. Patients in the intensive care unit (ICU) are known to be at elevated risk for VTE [1]. Previous study showed that 2.7% of patients had proximal DVT on admission to the ICU, and an additional 9.6% developed DVT during their ICU stay [2]. VTE, especially PE, may seriously influence patients’ prognosis, prolong the length of stay, and increase mortality [3]. Significant increases have been found in both ICU and hospital mortality rates for patients not receiving thromboprophylaxis within 24 hours [4]. In view of this, a series of VTE prophylaxis guidelines have been developed; however, these guidelines have not been properly implemented and are extremely underutilized [3]. Thus, it is necessary to explore how to effectively integrate VTE prophylaxis evidence into clinical practice.

The implementation of evidence into practice is a proactive and gradual process that differs from the promotion of certain technologies through visualization tools to persuade the target population to make a purchase. Innovations in the healthcare sector are complex [5]. Before an innovation is implemented, the factors that influence the innovation need to be clearly identified. Expertise and skills are needed to facilitate clinical decision-making to enable the implementation of the innovation in a particular clinical setting. A systematic review of the literature on healthcare reforms by Greenhalgh et al. [6] revealed that there are currently five major challenges in the implementation of evidence; evidence of innovation(E-1); healthcare workers’ need to learn about new evidence(C-2); the organizational structure and functions of healthcare institutions(C-3); factors concerning patients (including personal and family factors) (C-4); and evidence-related communication and facilitation(F-5). When developing measures and strategies related to evidence implementation (EI), the above five factors should be considered in accordance with the preferences of the target population [7]. These challenges coincide with the core concept of promoting the functional relationship between Evidence (E-1), Context (C-234) and Facilitation (F-5) in the Promoting Action on Research Implementation in Health Service (PARIHS) framework [8–10]. The PARIHS conceptual framework, proposed in 1998, suggests that the successful implementation (SI) of evidence into practice depends on the quality and type of Evidence, Context and Facilitation [SI = f (E, C, F)] and emphasizes that these three factors are of equal importance in evidence implementation (EI) [9]. This framework has been widely used in evidence-based practice, and a number of limitations to its effective utilization have been identified [11]. These mainly include failure to address key dimensions [12–14], failure to acknowledge the central role of individuals in the implementation process [15], and the development of relevant implementation theories [16–18]. Therefore, in 2016, Kitson et al. [11,19] combined the application and development of the PARIHS conceptual framework over the previous 20 years and updated it as the integrated-Promoting Action on Research Implementation in Health Service (i-PARIHS) framework. The three-matrix framework of the three core elements (evidence, context and facilitation) was transformed into a practical spiral-shaped structural framework. The i-PARIHS framework as a continuous spiral which starts with a focus on the innovation and the recipients, moving out to the different layers of context (inner context at local and organizational level and outer context at wider system and policy level. The focus on elements represented by each round of coils and the actions taken by facilitators are illustrated in words, implying that the SI of evidence requires the combination of innovation, recipients, context, and facilitation for the assessment, adjustment and integration of the other three elements, namely, SI = Fac^n^ (I+R+C) [20]. In the revised version, facilitation is considered the active ingredient of implementation and is achieved via a network of facilitators who apply a variety of skills and strategies to formulate and improve the implementation process, develop and manage relationships with key stakeholders, and identify and overcome barriers to implementation within the context [11].

This study takes the clinical implementation of VTE nursing evidence in two ICU units of a hospital in Suzhou from May to September 2017 as an example and describes how to integrate the "best" evidence into clinical VTE nursing in the ICU under the guidance of the i-PARIHS framework.

## Methods

**Fig 1:**
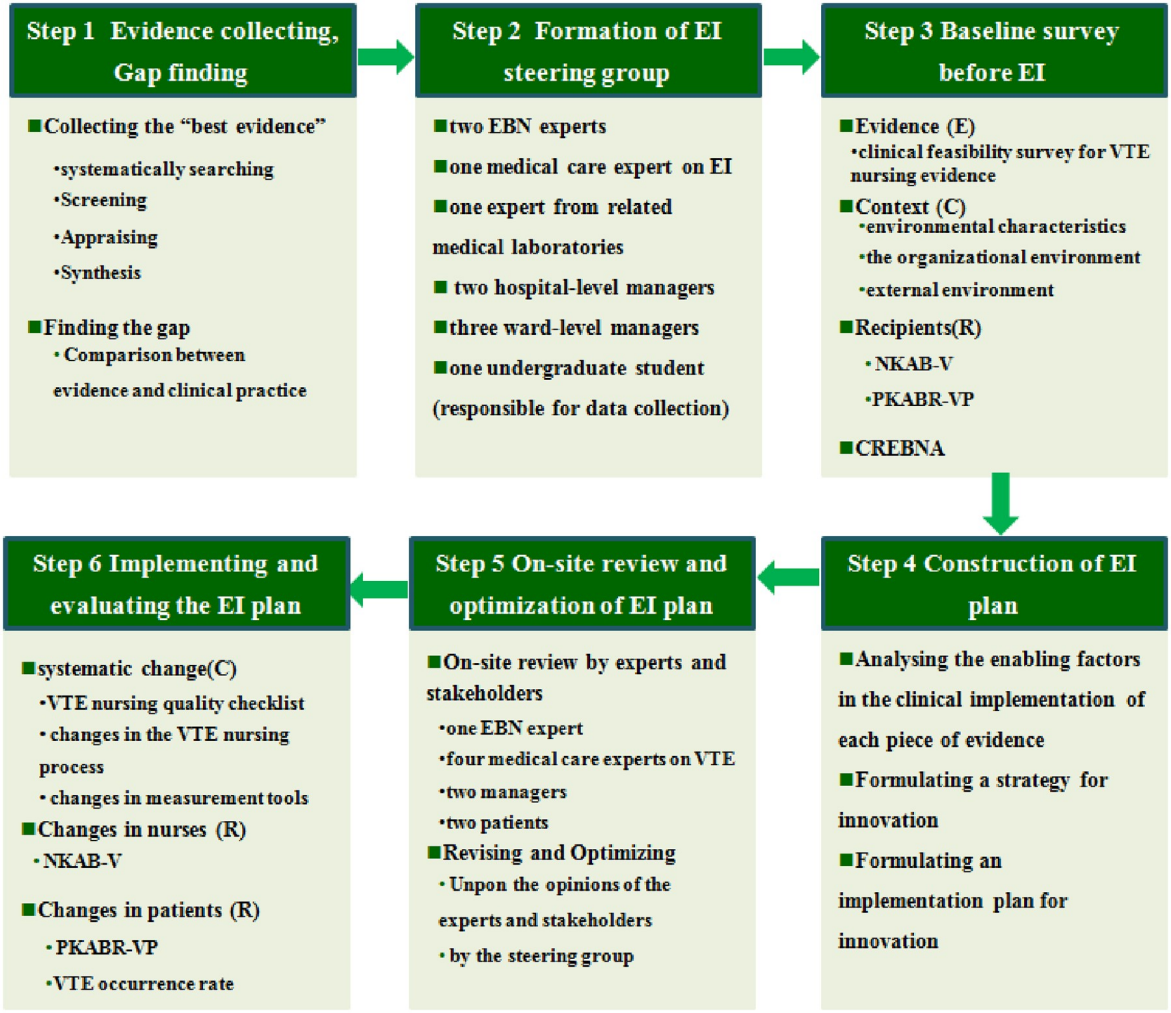
The structure of evidence implementation process. EBN: evidence-based nursing; EI: evidence implementation; VTE: venous thrombo-embolism; NKAB-V: nurses’ knowledge, attitudes and behaviors regarding VTE nursing; KABR-VP: patients’ knowledge, attitudes and behaviors regarding VTE nursing; CREBNA: the clinical readiness of evidence-based nursing assessment.

An implementation-study design was used in this study because it provides a practical method of understanding multiple perspectives, different types of causal pathways, and multiple types of outcomes as accepted features of implementation research problems [21,22]. This study obtained ethical approval from the Medical Ethics Committee of the First People’s Hospital of Kunshan (id: 2018–01). The EI under discussion was to be conducted in the Comprehensive ICU (Unit A) and Neurological ICU (Unit B) of the First People’s Hospital of Kunshan (see Table 1), which had similar VTE occurrence rates in the past year and volunteered to participant in the EI program. All participants signed the informed consent form. The process of integrating evidence into practice in this study included six steps (see Fig 1).

**Table 1:** General condition of the two included ICUs.

Characteristic	Comprehensive ICU (Unit A)	Neurological ICU (Unit B)
Authorized beds (n)	20	18
Monthly turnover cases (n)	60	50
Average length of stay (n)	10.2	8.5
Main cases	all types of critically ill patients	Patients with cerebral hemorrhage/cerebral infarction
Number of nurses	36	11

### Pre-Evidence Implementation (EI) assessment

Based on the i-PARIHS framework, a baseline survey programme was developed to clarify the characteristics of the core elements of this framework, i.e., evidence, context and recipients from 10^th^ to 25^th^ May 2017.

#### Evidence

A clinical feasibility survey for VTE nursing evidence was conducted to clarify whether “innovation” or the “best” evidence to be implemented was ethically and culturally appropriate, operable, and acceptable with respect to economic cost and safety during the clinical implementation process. This survey tool was self-designed using a four-point Likert scale (1–4) to assess the items’ ethical and cultural appropriateness, operability, and acceptability with respect to economic cost and safety, with 1 indicating the lowest degree (e.g., the lowest appropriateness) and 4 indicating the highest degree (e.g., the highest appropriateness). Higher scores signify a higher degree of feasibility [23]. The participating medical professionals were requested to complete the survey based on their professional judgement.

#### Context

Spot inspection and communicating with the nurses were used to understand the characteristics of “context”, including (i) environmental characteristics: the configuration of human, financial and material resources; (ii) the organizational environment: team culture (whether the team is good at accepting “innovation”) and leadership; and (iii) external environment: support from related departments. The clinical readiness for evidence-based nursing assessment (CREBNA) was used to assess the clinical readiness for evidence-based nursing (EBN). The CREBNA scale included 3 dimensions (evidence, context and facilitation) and 31 items using a five-point scale (1–5): 1, completely unmatched; 2, basically unmatched; 3, partially matched; 4, basically matched; and 5, completely matched. Higher scores signify a greater level of clinical readiness for EBN. The content validity index (CVI) of the scale was 0.976, and the Cronbach’s α coefficient was 0.959 for the total scale and 0.940, 0.933, and 0.915 for the above three subscales, respectively [24]. The test-retest reliability was 0.917, and the split-half reliability coefficient was 0.978 for the total scale [25].

#### Recipients

The innovation “recipients” included healthcare personnel, patients, and caregivers on the EI site.

#### Healthcare personnel

A survey of nurses’ knowledge, attitudes and behaviours regarding VTE nursing (NKAB-V) as well as medical professionals’ compliance with VTE nursing evidence was conducted to clarify the characteristics of healthcare personnel.

NKAB-V was a 22-item scale developed based on the VTE nursing evidence to be implemented [23]. The 3-point scale was reviewed by experts in evidence-based practice (EBP) and VTE nursing, and its CVI was 0.96, indicating that this scale had good content validity [26].

The survey format assessing the compliance of medical professionals with EBP was developed according to the evidence items of VTE nursing and was implemented accordingly. In this format, “YES” (observer’s EBP behaviour conforms to the recommendation of evidence items) and “NO” (observer’s EBP behaviour does not conform to the recommendation of evidence items) were used as the evaluation criteria. The compliance of the medical professionals with EBP regarding VTE nursing was calculated as the number of items rated “YES” divided by total EI numbers of VTE nursing within the EI period [23].

Participants in these surveys included the nurses of the two ICUs who had worked in the pilot units for at least one year and were willing to participate in these surveys. A purposive and accessible sample of doctors who were at least associate chief physicians and had worked in the ICU for at least 10 years was included.

#### Patients

Patients’ knowledge, attitudes and behaviors regarding VTE nursing (PKABR-VP) and VTE occurrence rate were obtained to conduct the baseline survey. The development of PKABR-VP was similar to that of NKAB-V, and its CVI was 0.98. Colour Doppler Ultrasound was used to determine whether the patient had VTE when they were admitted to and discharged from the ICU.

The questionnaires were handed out and reclaimed on the spot. The compliance of the medical professional with EBP regarding VTE nursing was evaluated by field observation with reference to medical records and interviews with observers. The scaling of each item was ordinal; therefore, in this study, the constituent ratio was used to describe the results instead of the mean and standard deviation. According to the Pareto principle (also known as the 80/20 rule) [27], when analyzing the results, we focused on the items of which the constituent ratio was below 80%. For example, in the clinical feasibility survey, when an item received a score with a constituent ratio below 80%, it was marked and discussed in the subsequent focus group interview to analyze the causes, influencing factors and interventions involved in changing the condition.

### Construction, on-site expert review and optimization of the Evidence Implementation (EI) plan

Based on the results of the baseline survey, the steering group analyzed the enabling factors in the clinical implementation of each piece of evidence and formulated a viable innovation strategy. Additionally, factors enabling EI were analyzed from the perspectives of “innovation,” “recipients” and “context” to provide a reference for pre-implementation targeted training (e.g., EI knowledge, tools, processes, and operating skills). There was a consensus process for the development of innovation strategies. The strategies were discussed and tailored by the steering group, and were presented in the S1–S5 Files.

For example, during the clinical feasibility survey, the constituent ratio of the operability of daily using intermittent pneumatic compression (IPC) was below 80%. When analyzing the causes, we found that there were no sufficient IPC pumps in the two ICUs for implementation. Then the two hospital-level managers and three ward-level managers included in the steering group were responsible for determining whether the facility had the budget to purchase this equipment, the length of the purchasing cycle, and whether the purchasing cycle would affect clinical implementation. All of these were presented in the focus group discussion on the formation of the innovation strategies. In addition, the economic efficiency of IPC was also considered for the consensus process of innovation strategies.

The initial EI plan for VTE nursing evidence developed by the steering group. Then one EBN expert, four medical care experts on VTE, two managers, and two patients recruited using purposive sampling were invited to participate in a group interview to discuss the scientific rigor, feasibility, and safety of the plan. After the interview, their opinions were sorted and ranked in descending order according to consensus degree, and the proposal with the greatest consensus was given the highest priority, upon which the clinical EI plan was revised and optimized by the steering group.

### Post-EI evaluation

During the implementation of the EI plan from 15^th^ July to 20^th^ Sept. 2017, members of the steering group conducted rounds in the units periodically, addressed challenges, and collected feedback and suggestions proposed by the professionals at the research site. The feedback and suggestions were sorted, and common problems were analysed to determine whether specific training was required.

#### Systematic changes

The systematic change was assessed with regard to a VTE nursing quality checklist, changes in the VTE nursing process, and measurement tools.

#### Changes in nurses

Changes in nurses were evaluated using NKAB-V.

#### Changes in patients

Changes in patients were evaluated using PKABR-VP and VTE occurrence rate.

### Data analysis

Analyses were performed by IBM SPSS Statistics 24. For the pre- and post-EI evaluation, descriptive statistics were used to analyze the quantitative data related to the outcomes regarding the context, nurses and patients. To analyse the scale of knowledge, attitudes and behaviours regarding VTE nursing of the nurses and patients, we calculated the total scores for each domain and compared the difference between the two ICUs pre- and post-implementation using *t* tests. Additionally, the ratio of VTE was compared between the two ICUs throughout the EI using *t* tests.

## Results

### Baseline survey for VTE nursing evidence

A total of 47 nurses and 5 doctors from the hospital’s comprehensive ICU and neurology ICU participated in the survey of clinical feasibility and CREBNA, and the included nurses also completed the NKAB-V survey. The general participant information is shown in Table 2.

**Table 2:** General information of ICU medical staff participating in clinical feasibility survey of VTE nursing evidence.

Items	Doctors	Nurses
Age (years) (mean ±SD)	43.00±7.42	30.85±4.31
Gender n(%)		
Female	0	40(76.92)
Male	5(9.61)	7(13.46)
Level of Education		
Postgraduate	2(3.85)	2(3.85)
Undergraduate	3(5.76)	40(76.92)
Junior College	0	5(9.61)
Technical Secondary School	0	0
Professional Titles		
Senior	1(1.92)	0
Deputy senior	4(7.69)	4(7.69)
Middle	0	15(28.85)
Primary	0	28(53.85)
Years of Working		
<5	0	4(7.69)
5~10	0	22(42.31)
>10	5(9.61)	28(53.85)

**Fig 2:**
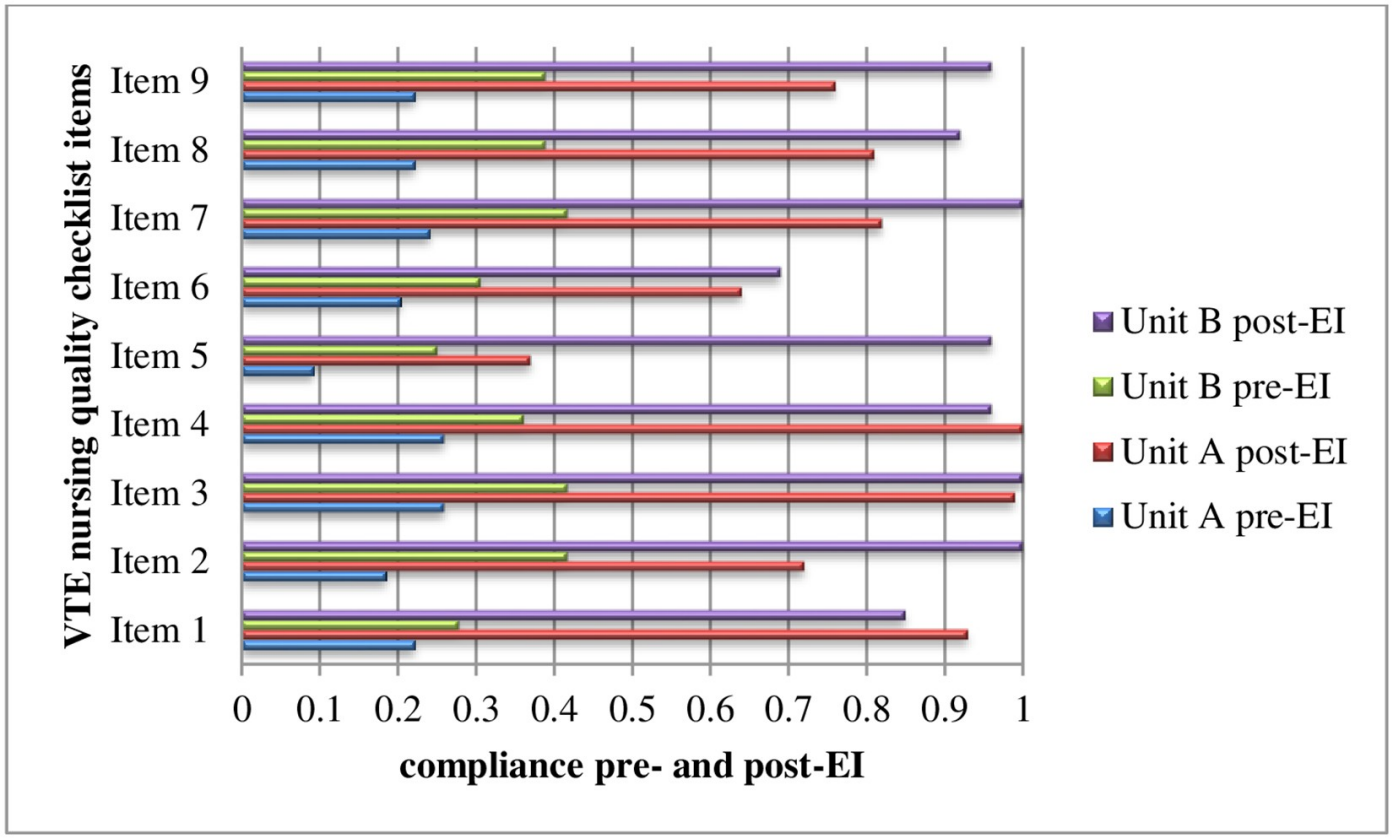
Nurses’ compliance with VTE nursing quality checklist items pre- and post-EI.

In the survey of clinical feasibility, the constituent ratio of scoring 4 was below 80% for all of the following items: the operability of assessing the risk of haemorrhage in patients receiving anticoagulant therapy each shift, passive exercise, assessing the contraindications of intermittent pneumatic compression (IPC), and daily use of IPC, specifically, the acceptability of the economic cost of assessing the contraindications of IPC, and the daily use of IPC, and the appropriateness and safety of the IPC. The results of the CREBNA showed that the constituent ratio of scoring 4 or 5 was below 80% in the dimension of evidence for “*screening of evidence fully takes into account current medical conditions and medical standards*,” “*the evidence solves problems within the scope of medical/nursing duties*, *and corresponding interventions can be conducted*,” and “*evidence has been transformed into forms that are easy to disseminate*, *understand and apply*, *such as procedures*, *practice manuals*, *programme posters*, *etc*.,*”* in the dimension of context for “*nurse managers can reasonably allocate human resources according to clinical work*,” “*I have good execution of the tasks assigned by superiors*” and “*the ward I am in has a multi-disciplinary cooperation culture and workflow*,” and in the dimension of facilitation for “*I have the opportunity to participate in decision-making on ward-related matters (development/change of workflow*, *resource allocation*, *staffing*, *etc*.*)*,” “*the ward I am in has the information resources (medical data*, *software development technology*, *technical staff support*, *etc*.*) needed to carry out evidence-based practice*” and “*there are plans to promote evidence-based practice (to disseminate current evidence to other hospitals/wards)*.” The results of the NKAB-V indicated that the included ICU nurses’ knowledge, attitudes and behaviours regarding VTE nursing were poor. In the compliance survey of the VTE nursing evidence to be implemented, the implementation rates of each item in the two ICUs were all below 50% (see Fig 2).

### Systematic changes

Systematic change mainly included the following: developing a VTE nursing process, adding the VTE nursing indicator into the nursing record sheet, embedding the Caprini scaling and the corresponding nursing into the hospital information system (HIS), formulating the health education sheet of VTE for patients, and establishing the VTE nursing quality checklist. Compared with the baseline survey, nurses’ compliance with each piece of evidence improved significantly in both units A and B, with greater improvement in the latter than the former (see Fig 2).

### Changes in nurses

Thirty nurses from unit A and unit B completed the pre- and post-EI NKAB-V survey. Before EI, standard screening, assessment and intervention of VTE had not been conducted in either unit A or unit B. There was no significant difference in the attitude scores of VTE nursing between the two units. However, the VTE nursing knowledge scoring of unit A was higher than that of unit B, while the behaviour scoring for VTE nursing of unit B was higher than that of unit A. After EI, the knowledge scoring for VTE nursing increased in the two units compared with the baseline; the improvement in unit A was statistically higher than that of unit B. There was no significant difference in the attitude score of VTE nursing between the two units (see Table 3). Regarding the implementation of VTE nursing, there was no significant difference in the following items: item 15 (Nurses should be proactive in educating patients about preventive measures against VTE), item 18 (Nurses should assess the risk of haemorrhage for patients undergoing anticoagulant therapy in each shift), item 20 (Nurses should provide timely health education for patients with haemorrhage risk), item 21 (Nurses can instruct patients to adopt appropriate physical prevention in time based on the patient’s condition) and item 22 (Nurses should monitor patients closely for adverse effects such as haemorrhage when patients are taking anticoagulants). Regarding the scores of the remaining 17 items, unit B was statistically higher than unit A.

**Table 3:** NKAB-V scaling pre- and post-EI in the/ two units.

Items	Group	Pre-EI (mean±SD)	Post-EI (mean±SD)	t	p
Total Score of Knowledge	Unit A	52.25±7.19	64.71±2.49	-8.46	<0.01
	Unit B	43.00±2.45	70.83±11.84	-5.88	<0.01
	t	3.07	2.44		
	p	<0.01	0.02		
Total Score of Attitude	Unit A	50.25±7.46	64.51±2.87	-9.33	<0.01
	Unit B	44.50±2.95	59.33±3.67	-7.27	<0.01
	t	1.83	3.76		
	p	0.08	<0.01		
Total Score of Behavior	Unit A	50.96±6.89	60.46±4.25	-7.28	<0.01
	Unit B	45.17±2.86	55.33±6.89	-4.09	<0.01
	t	1.99	2.33		
	p	0.06	0.03		

* NKAB-V: nurses’ knowledge, attitudes and behaviors regarding VTE nursing

### Changes in patients

Twenty patients from unit A and eighteen patients from unit B completed the pre- and post-implementation PKABR-VP survey. After the EI, their knowledge, attitude and behaviour scores regarding VTE self-care all improved compared with the baseline (see Table 4). During the EI, a total of 100 patients were admitted to the two ICUs, and there was no VTE occurrence.

**Table 4:** Changes of PKABR-VP scaling pre- and post-EI.

Variation	N	Pre-EI (mean±SD)	Post-EI (mean±SD)	*t*	*P*
Total Score of Knowledge	38	18.37±7.34	28.61±1.35	8.24	<0.01
Total Score of Attitude	38	18.26±7.63	28.42±1.13	7.95	<0.01
Total Score of Behavior	38	19.92±8.79	29.18±1.09	6.28	<0.01

* PKABR-VP: Patients’ Knowledge, Attitudes and Behaviors scaling regarding VTE Prophylaxis

## Discussion

In this study, we used evidence implementation (EI) process of the venous thromboembolism (VTE) nursing as an example and described how to make the EI process achievable by developing a clinical EI plan.

Differences in the medical technology level and medical environment make it impossible to ensure that the evidence, even if it is the “best” available, can be implemented in all clinical settings [28]. During this study, several items of the “best” evidence on VTE nursing got lower scaling in the clinical feasibility survey due to the limitation of the facilities, poor knowledge, attitudes and skills of healthcare personnel. Through EI, those evidences included in the EI plan have been successfully incorporated into the routine care of VTE management by developing a series of VTE nursing standards and tools, which has changed the professional behaviours of the nurses in the two ICUs regarding VTE nursing.

The implementation of innovation ultimately depends on the individuals in the organization, and the quality of the implementation process ultimately depends on the daily work quality of these individuals. After EI, Nurses’ knowledge, attitudes and behaviours regarding VTE nursing (NKAB-V) in the two ICUs have significantly improved compared with baseline survey. The scoring of the knowledge dimension of NKAB-V in Unit B was higher than that of Unit A, while the scorings of the attitudes and behaviours dimension in Unit B were lower than those of Unit A. However, the compliance with each piece of evidence in Unit B was higher than Unit A except item 1 and item 4. Considering NKAB-V is a self-report scale, which may result in certain bias, and the compliance of the nurses were assessed by the ward managers, there may exist differences between the two evaluations, even though they both promote the professional growth of the nurses in this study to some extent. In addition, some issues should be given more attention to guarantee the successful implementation of evidence. “Opinion leaders” should be identified in the pilot wards [29]. The opinions and behaviours of such healthcare workers are often easily accepted and followed by other medical personnel, so it is important to convince and train them because they are key players in leading and advancing EI. This view is in accordance with the health promotion theory that “it is easier for information disseminators and change agents within the target population to achieve the success of innovation” [6]. “Core figures,” such as frontline ward managers, “opinion leaders,” and nurse leaders, should be incorporated into the nursing procedure reform with the goal of improving their compliance with innovation.

The ultimate objective of the EI program is to improve the patients’ clinical outcomes. During this study, patients’ knowledge, attitudes and behaviours regarding VTE nursing have been significantly promoted compared with the baseline survey, which may influence the patients’ self-management of VTE to some extent, resulting in better clinical outcomes regarding VTE occurrence.

In summary, constructing the clinical implementation plan for the “best” evidence falls under the preparation phase. The method used to screen the “prevalent” or “most needed” “best” evidence is the first decision to be considered by each researcher or EI facilitator; that is, the starting point for EI is based on clinical problems that need to be solved or improved rather than the individual interests of the researcher. It is necessary to correctly understand “to what degree evidence and policies of the EI site, practices and priorities match” [30]. Whether the evidence can meet the main objectives of organizational development at the current stage will certainly affect the smooth implementation of the evidence in clinical practice [31]. Therefore, it is crucial to accurately analyze the enabling and hindering factors in context to ensure the smooth implementation of evidence. Context in the i-PARIHS framework includes inner context: local and inner context: organization as well as outer context [19]. In the EI process, we tend to pay more attention to the enabling and hindering factors in the first two contexts while ignoring the impact of the outer context on the entire EI process. The characteristics of outer context include, in a broad sense, policy drivers and cultural customs, which may affect the initiative, incentives, regulatory systems, networks and relationships between different organizations in inner context: local [32]. Therefore, when implementing evidence, changes in the macro medical environment (especially newly introduced national policies and regulations), specific regional cultures, and people’s values that are associated with the EI should be fully considered.

Due to limitations in time and funding, the EBP was only implemented in two units, and the clinical implementation of evidence continued for only two months, covering a limited number of patients. In future research, a multi-centre evidence implementation with a longer duration and outcome observations should be performed to examine the long-term application effects of the EBP project.

## Conclusion

The updated i-PARIHS framework is more descriptive of the key concepts that affect EI than PARIHS and may provide more instructive guidance for incorporating evidence into practice. The EI programme of incorporating the “best” evidence on VTE nursing into clinical practice using the i-PARIHS framework has been successfully conducted in the clinical practice, and evidences implemented have been successfully incorporated into the routine care of VTE, so this EI program could be used as a reference for similar clinical utilizations.

## Implications

Three key points were identified with regard to the EI: (i) the need to simplify the innovation strategies as much as possible to promote their operability; (ii) the need for close attention by hospital administrators (including the president, head of the nursing department, the department director and the head nurse) to the EI can facilitate the EI process effectively; (iii) after the EI programme, making the well-integrated evidence part of the standards for routine care can promote the sustainability of the EI.

## Supporting information

S1 File Standards for reporting implementation studies: The StaRI checklist for completion. (DOCX)

S2 File Assessment scale for evidence-based practice preparation. (DOCX)

S3 File Clinical feasibility survey for VTE nursing evidence. (DOCX)

S4 File ICU nurses’ knowledge, attitudes and behaviors regarding VTE nursing. (DOCX)

S5 File VTE nursing quality checklist. (DOCX)

## Abbreviations

CREBNA: clinic readiness of evidence-based nursing assessment
CVI: content validity index
DVT: deep venous thrombosis
EBN: evidence-based nursing
EI: evidence implementation
ICU: intensive care unit
i-PARIHS: integrated-promoting action on research implementation in health services
IPC: intermittent pneumatic compression; HIS: hospital information system
NKAB-V: nurses’ knowledge, attitudes and behaviours regarding VTE nursing
PE: pulmonary embolus
PKABR-VP: patients’ knowledge, attitudes and behaviours regarding VTE nursing
VTE: venous thromboembolism
