# Supplementary material for: Integrating the “best” evidence into nursing of venous thromboembolism in ICU patients using the i-PARIHS framework
Source: PLoS One. 2020 Aug 6;15(8):e0237342. doi: 10.1371/journal.pone.0237342 (PMC7410309; doi:10.1371/journal.pone.0237342)
Supplement: S2 File — (DOCX) [file pone.0237342.s002.docx]

**Questionnaire No.: _____**

**Assessment Scale for Evidence-based Practice Preparation**

Instructions: This survey aims to assess the preparation of evidence-based practice in your ward, to identify the obstacles and facilitatimg factors, in order to ensure the smooth progress of evidence-based practice. The questionnaire is divided into two parts. Part I is the basic information, and Part II is the scale. Please read each item carefully and select the option of which the contents match the actual situation, 1= do not match at all, 2= basically do not match, 3= partly match, 4= basically match, 5= completely match.

1. your sex: 2. the year of your birth:

3. your degree / Degree:

Technical secondary school; junior college; Bachelor degree (including reading); master's degree (included in reading);

4. your working life: year; your working life in this college: year.

5. your position / position is:

The Director / deputy director of nursing department; nurse head; specialist nurse; nurse; others.

6. your title is:

Primary (Division); junior (Division); intermediate; advanced (sub high); advanced (high).

8. your research experience: No;

If so, the type of research you undertake is (multiple options):

Investigation or descriptive research; experimental research; experimental research; qualitative research;

**Part I: Basic Information**

1. Gender: Male□ Female□

2. Date of Birth: ＿＿＿＿＿

3. Education Background:

□ Technical Secondary School; □ Junior College; □ Bachelor’s Degree (candidates included ) □ Master's Degree (candidates included); □ Doctoral Degree (candidates included)

4. Working Years: _____ years; Working Years in This Specialty: _____ years

5. Position/post: ＿＿＿＿＿

□ Director/Deputy Director of Nursing Department; □ Head Nurse; □ Nurse Specialist; □ Nurse; □ Others _____

6. Professional Title:

□ junior (nurse); □ junior (primary nurse); □ intermediate; □ senior (sub-senior); □ senior

7. Research Experience: □ No; □ Yes

If your answer is “Yes”, the type of research you undertake is (multiple choices):

□Survey or descriptive research; □Quasi-experimental research; □Experimental research; □Qualitative research; □ Others

8. Knowledge of evidence-based practice / evidence-based nursing practice / evidence implementation:

□ I haven't heard about it. □ I've heard about it, but I don't know about it. □ I have some knowledge about it. □ I know quite a lot about it. □ I am very familiar with it.

9. Have you organized or participated in evidence-based practice / evidence implementation programs? □ No; □ Yes

10. Do you think it is necessary to carry out evidence-based practice / evidence implementation programs? □ No; □ Yes

If your answer is “Yes”, what do you think is the significance of conducting evidence-based practice / evidence implementation programs? (multiple choices)

□ Upgrade the quality of nursing care; □ Save medical costs; □ Improve patient satisfaction;

□ Meet the needs of discipline development; □ Others ________

**Part II Formal Survey**

**1. Evidence Subscale**

**The statements in the following items are for the evidence-based practice plan that is to be implemented.**

| Items | Completely match | Basically match | Partly match | Basically do not match | Do not match at all |
| --- | --- | --- | --- | --- | --- |
| 1. The source of evidence is reliable  Note: “Evidence” specifically refers to the standards/processes that are introduced into clinical practice in our evidence-based practice program. | 5 | 4 | 3 | 2 | 1 |
| 2. The evidence is evaluated through a rigorous quality assessment process. | 5 | 4 | 3 | 2 | 1 |
| 3. The evidence is appropriate for patients/medical staff in institutions that are about to implement evidence-based practice programs. | 5 | 4 | 3 | 2 | 1 |
| 4. Screening of evidence combines the work experience and professional judgment of clinical nurses. | 5 | 4 | 3 | 2 | 1 |
| 5. Screening of evidence takes into account the needs of patients. | 5 | 4 | 3 | 2 | 1 |
| 6. Implementation of the evidence can promote the rehabilitation of patients and directly or indirectly improve the outcome of patients. | 5 | 4 | 3 | 2 | 1 |
| 7. Implementation of the evidence can improve the quality of medical/nursing care. | 5 | 4 | 3 | 2 | 1 |
| 8. Evidence does not violate national policies and laws. | 5 | 4 | 3 | 2 | 1 |
| 9. Screening of evidence fully takes into account current medical conditions and medical standards. | 5 | 4 | 3 | 2 | 1 |
| 10. The evidence solves problems within the scope of medical/nursing duties, which can be intervened correspondingly. | 5 | 4 | 3 | 2 | 1 |
| 11. I am happy to accept the evidence that is to be implemented clinically, which is in line with my self-requirements and values. | 5 | 4 | 3 | 2 | 1 |
| 12. Evidence has been transformed into forms that are easy to disseminate, understand and apply, such as procedures, practice manuals, program posters, etc. | 5 | 4 | 3 | 2 | 1 |

**2. Context Subscale**

**The following items are evaluated for the ward/organization/institution where the evidence-based practice is about to be implemented**

| Items | Completely match | Basically match | Partly match | Basically do not match | Do not match at all |
| --- | --- | --- | --- | --- | --- |
| 1. Nurse managers are good at actively exploring and improving clinical work.  Note: The nurse manager refers to the person who directly manages us in the ward in question and has certain authority. If you are the nurse manager yourself, then this form is for self-evaluation. | 5 | 4 | 3 | 2 | 1 |
| 2. The nurse manager has much influence over us and we are willing to execute her/his advice or orders. | 5 | 4 | 3 | 2 | 1 |
| 3. Nurse managers can reasonably allocate human resources according to clinical work. | 5 | 4 | 3 | 2 | 1 |
| 4. Nurse managers have good communication and coordination skills | 5 | 4 | 3 | 2 | 1 |
| 5. Nurse managers are fully open to our suggestions and opinions. | 5 | 4 | 3 | 2 | 1 |
| 6. I am willing to try new clinical nursing procedures, methods, techniques, etc. | 5 | 4 | 3 | 2 | 1 |
| 7. I have a good execution of the tasks assigned by the superiors. | 5 | 4 | 3 | 2 | 1 |
| 8. Our team members can work together to achieve specific goals. | 5 | 4 | 3 | 2 | 1 |
| 9. The ward I am in has a multi-disciplinary cooperation culture and workflow. | 5 | 4 | 3 | 2 | 1 |

**3. Facilitation Scale**

| Items | Completely match | Basically match | Partly match | Basically do not match | Do not match at all |
| --- | --- | --- | --- | --- | --- |
| 1. There are facilitators with extensive expertise and clinical experience in the evidence-based practice team.  Note: In practice, facilitators refer to one or more individuals who encourage, help, and guide us through the practice program. They facilitate the implementation of the entire evidence-based practice. | 5 | 4 | 3 | 2 | 1 |
| 2. There are facilitators who can develop practical evidence-based practice programs in the evidence-based practice team. | 5 | 4 | 3 | 2 | 1 |
| 3. The forthcoming evidence-based practice has incorporated all relevant personnel, e.g. researchers, doctors, nurses and other multidisciplinary team members. | 5 | 4 | 3 | 2 | 1 |
| 4. There are incentive policies, such as job prospects, learning opportunities, collective honors, rewards, etc. | 5 | 4 | 3 | 2 | 1 |
| 5. There are training courses in various forms related to evidence-based practice projects, such as lectures, video lectures, seminars, and drills. | 5 | 4 | 3 | 2 | 1 |
| 6. I have the opportunity to participate in decision-making on ward-related matters (development/change of workflow, resource allocation, staffing, etc.) | 5 | 4 | 3 | 2 | 1 |
| 7. The forthcoming evidence-based practice program has gained support from the management (hospital/nursing department). | 5 | 4 | 3 | 2 | 1 |
| 8. The ward I am in has the information resources (medical data, software development technology, technical staff support, etc.) needed to carry out evidence-based practice. | 5 | 4 | 3 | 2 | 1 |
| 9. There is a feedback system to optimize practice based on feedbacks from clinical nurses and patients. | 5 | 4 | 3 | 2 | 1 |
| 10. There are plans to promote the evidence-based practice (to disseminate current evidence to other hospitals/wards). | 5 | 4 | 3 | 2 | 1 |
