# Supplementary material for: Integrating the “best” evidence into nursing of venous thromboembolism in ICU patients using the i-PARIHS framework
Source: PLoS One. 2020 Aug 6;15(8):e0237342. doi: 10.1371/journal.pone.0237342 (PMC7410309; doi:10.1371/journal.pone.0237342)
Supplement: S3 File — (DOCX) [file pone.0237342.s003.docx]

**Clinical feasibility survey for VTE nursing evidence**

**Instructions:**

The following items are to assess the ethical and cultural appropriateness, operability, acceptability of economic cost and safety of each VTE nursing item to be implemented, using a four-point Likert scale (1-4), 1: lowest degree, e.g. the lowest appropriateness, 4: highest degree, e.g. the highest appropriateness. There is no true or false regarding the answer, and you need to fill out the form according to the facts. If you have any suggestion or advice, please fill in the rightmost column.

| No. | Item for Evaluation | Ethical and cultural appropriateness | Operability | Acceptability of economic cost | Safety | Your  Suggestion |
| --- | --- | --- | --- | --- | --- | --- |
| 1 | VTE risk assessment should be completed using the Caprini scale within 24 hours of the patient's ICU admission |  |  |  |  |  |
| 2 | For patients undergoing anticoagulant therapy, risk for hemorrhage should be assessed in each shift |  |  |  |  |  |
| 3 | When the patient's clinical condition changes, risk for hemorrhage and VTE should be reassessed |  |  |  |  |  |
| 4 | Document and analyze the patient's intake and output in each shift to prevent dehydration |  |  |  |  |  |
| 5 | After the patient's condition becomes stable, passive movement of the lower extremities should be performed as soon as possible |  |  |  |  |  |
| 6 | Assess whether the patient has contraindications to IPC use (severe lower extremity arteriosclerotic hemorrhage, congestive heart failure, pulmonary edema, lower extremity DVT, thrombophlebitis, severe local lower extremity diseases such as dermatitis, gangrene, recent surgery and severe deformity). If not, IPC can be used according to the doctor’s orders |  |  |  |  |  |
| 7 | Use of IPC: 1 time/day, 2 hours/time |  |  |  |  |  |
| 8 | Since compression stockings cannot significantly lower the incidence of Post thrombotic Syndrome (PTS), its necessity should be assessed before use and each day during use with caution |  |  |  |  |  |
| 9 | For patients using compression stockings to prevent PTS, daily observation of whether the circumference of stockings fits the lower extremities should be done to avoid folding |  |  |  |  |  |
