# Supplementary material for: Integrating the “best” evidence into nursing of venous thromboembolism in ICU patients using the i-PARIHS framework
Source: PLoS One. 2020 Aug 6;15(8):e0237342. doi: 10.1371/journal.pone.0237342 (PMC7410309; doi:10.1371/journal.pone.0237342)
Supplement: S4 File — (DOCX) [file pone.0237342.s004.docx]

**ICU Nurses’ knowledge, attitudes and behaviors**

**regarding VTE nursing**

Thanks for your participation in this survey of your knowledge, attitudes and behaviors regarding VTE nursing. Please tick (“√”) the box preceding the option that most fits you.

1. Date of birth:________(month)/________(year)

2. When you came to work in the current ward: ________(month)/________(year)

3.Your current education background:

□postgraduate □university □junior college □technical secondary school

4. Your current professional titles:

□chief superintendent nurse □co-chief superintendent nurse □nurse-in-charge □senior nurse □nurse

5.Your current position: □head nurse □nurse preceptor □nurse

| **No.** | **Item** |
| --- | --- |
| **1** | **ICU patient is a high risk group of venous thromboembolism (VTE).** |
|  | □3= fully aware □2= partly aware □ 1= unaware |
|  | □ 3=very important □ 2=relatively important □ 1=unimportant |
|  | □ 3=often consider □ 2=sometimes consider □ 1=never consider |
| **2** | **The mechanism of VTE** |
|  | □3= fully aware □2= partly aware □ 1= unaware |
|  | □ 3=very important □ 2=relatively important □ 1=unimportant |
|  | □ 3=often consider □ 2=sometimes consider □ 1=never consider |
| **3** | **The risk factors of VTE** |
|  | □3= fully aware □2= partly aware □ 1= unaware |
|  | □ 3=very important □ 2=relatively important □ 1=unimportant |
|  | □ 3=often consider □ 2=sometimes consider □ 1=never consider |
| **4** | **The clinical manifestations of VTE** |
|  | □3= fully aware □2= partly aware □ 1= unaware |
|  | □ 3=very important □ 2=relatively important □ 1=unimportant |
|  | □ 3=often consider □ 2=sometimes consider □ 1=never consider |
| **5** | **The preventive measures against VTE** |
|  | □3= fully aware □2= partly aware □ 1= unaware |
|  | □ 3=very important □ 2=relatively important □ 1=unimportant |
|  | □ 3=often consider □ 2=sometimes consider □ 1=never consider |
| **6** | **The importance of early identification of VTE** |
|  | □3= fully aware □2= partly aware □ 1= unaware |
|  | □ 3=very important □ 2=relatively important □ 1=unimportant |
|  | □ 3=often consider □ 2=sometimes consider □ 1=never consider |
| **7** | **The screening and assessment of VTE** |
|  | □3= fully aware □2= partly aware □ 1= unaware |
|  | □ 3=very important □ 2=relatively important □ 1=unimportant |
|  | □ 3=often consider □ 2=sometimes consider □ 1=never consider |
| **8** | **ICU nurses should assess the risk of VTE using Caprini risk assessment scale.** |
|  | □3= fully aware □2= partly aware □ 1= unaware |
|  | □ 3=very important □ 2=relatively important □ 1=unimportant |
|  | □ 3=do completely □ 2=sometimes do □ 1=never do  Reasons for “not do completely”：□increase workload □there’s no difference between doing and not doing □do it when it just come into the mind □other:____________ |
| **9** | **VTE is preventable.** |
|  | □3= fully aware □2= partly aware □ 1= unaware |
|  | □ 3=very important □ 2=relatively important □ 1=unimportant |
|  | □ 3=often consider □ 2=sometimes consider □ 1=never consider |
| **10** | **Nurses play an important role in VTE prevention.** |
|  | □3= fully aware □2= partly aware □ 1= unaware |
|  | □ 3=very important □ 2=relatively important □ 1=unimportant |
|  | □ 3=often consider □ 2=sometimes consider □ 1=never consider |
| **11** | **Nurses should assess the risk of VTE actively.** |
|  | □3= fully aware □2= partly aware □ 1= unaware |
|  | □ 3=very important □ 2=relatively important □ 1=unimportant |
|  | □ 3=do completely □ 2=sometimes do □ 1=never do  Reasons for “not do completely”：□increase workload □there’s no difference between doing and not doing □do it when it just come into the mind □other:____________ |
| **12** | **Nurses should implement the preventive measures against VTE.** |
|  | □3= fully aware □2= partly aware □ 1= unaware |
|  | □ 3=very important □ 2=relatively important □ 1=unimportant |
|  | □ 3=do completely □ 2=sometimes do □ 1=never do  Reasons for “not do completely”:□increase workload □there’s no difference between doing and not doing □do it when it just come into the mind □other:____________ |
| **13** | **Nurses should receive training on the prevention and treatment of VTE.** |
|  | □3= fully aware □2= partly aware □ 1= unaware |
|  | □ 3=very important □ 2=relatively important □ 1=unimportant |
|  | □ 3=often consider □ 2=sometimes consider □ 1=never consider |
| **14** | **The prevention of VTE should be included in the nursing quality audit.** |
|  | □3= fully aware □2= partly aware □ 1= unaware |
|  | □ 3=very important □ 2=relatively important □ 1=unimportant |
|  | □ 3=do completely □ 2=sometimes do □ 1=never do  Reasons for “not do completely”：□increase workload □there’s no difference between doing and not doing □do it when it just come into the mind □other:____________ |
| **15** | **Nurses should be proactive in educating the patients about the preventive measures against VTE.** |
|  | □3= fully aware □2= partly aware □ 1= unaware |
|  | □ 3=very important □ 2=relatively important □ 1=unimportant |
|  | □ 3=do completely □ 2=sometimes do □ 1=never do  Reasons for “not do completely”：□increase workload □there’s no difference between doing and not doing □do it when it just come into the mind □other:____________ |
| **16** | **Nurses should use the Caprini risk assessment scale correctly.** |
|  | □3= fully aware □2= partly aware □ 1= unaware |
|  | □ 3=very important □ 2=relatively important □ 1=unimportant |
|  | □ 3=do completely □ 2=sometimes do □ 1=never do  Reasons for “not do completely”：□increase workload □there’s no difference between doing and not doing □do it when it just come into the mind □other:____________ |
| **17** | **Nurses should complete the VTE risk assessment using the Caprini scale within 24 hours of the patient's ICU admission.** |
|  | □3= fully aware □2= partly aware □ 1= unaware |
|  | □ 3=very important □ 2=relatively important □ 1=unimportant |
|  | □ 3=do completely □ 2=sometimes do □ 1=never do  Reasons for “not do completely”：□increase workload □there’s no difference between doing and not doing □do it when it just come into the mind □other:____________ |
| **18** | **Nurses should assess the risk for hemorrhage for patients undergoing anticoagulant therapy in each shift.** |
|  | □3= fully aware □2= partly aware □ 1= unaware |
|  | □ 3=very important □ 2=relatively important □ 1=unimportant |
|  | □ 3=do completely □ 2=sometimes do □ 1=never do  Reasons for “not do completely”：□increase workload □there’s no difference between doing and not doing □do it when it just come into the mind □other:____________ |
| **19** | **Nurses should** **reassess the risk for hemorrhage and VTE when the patient’s clinical condition changes.** |
|  | □3= fully aware □2= partly aware □ 1= unaware |
|  | □ 3=very important □ 2=relatively important □ 1=unimportant |
|  | □ 3=do completely □ 2=sometimes do □ 1=never do  Reasons for “not do completely”：□increase workload □there’s no difference between doing and not doing □do it when it just come into the mind □other:____________ |
| **20** | **Nurses should provide timely health education for patients with hemorrhage risk.** |
|  | □3= fully aware □2= partly aware □ 1= unaware |
|  | □ 3=very important □ 2=relatively important □ 1=unimportant |
|  | □ 3=do completely □ 2=sometimes do □ 1=never do  Reasons for “not do completely”：□increase workload □there’s no difference between doing and not doing □do it when it just come into the mind □other:____________ |
| **21** | **Nurses can instruct the patients to adopt appropriate physical prevention in time upon the patients’ conditions.** |
|  | □3= fully aware □2= partly aware □ 1= unaware |
|  | □ 3=very important □ 2=relatively important □ 1=unimportant |
|  | □ 3=do completely □ 2=sometimes do □ 1=never do  Reasons for “not do completely”：□increase workload □there’s no difference between doing and not doing □do it when it just come into the mind □other:____________ |
| **22** | **Nurses should monitor the patients closely for adverse effects such as hemorrhage when the patients are taking anticoagulants.** |
|  | □3= fully aware □2= partly aware □ 1= unaware |
|  | □ 3=very important □ 2=relatively important □ 1=unimportant |
|  | □ 3=do completely □ 2=sometimes do □ 1=never do  Reasons for “not do completely”：□increase workload □there’s no difference between doing and not doing □do it when it just come into the mind □other:____________ |
