# Supplementary material for: Integrating the “best” evidence into nursing of venous thromboembolism in ICU patients using the i-PARIHS framework
Source: PLoS One. 2020 Aug 6;15(8):e0237342. doi: 10.1371/journal.pone.0237342 (PMC7410309; doi:10.1371/journal.pone.0237342)
Supplement: S5 File — (DOCX) [file pone.0237342.s005.docx]

**VTE nursing quality checklist**

| Ward: | Auditing date: _____/____/____ | Auditor: |
| --- | --- | --- |
| Admission No.: | Diagnosis: | VTE: □YES □NO |
| Occurrence of VTE: □Nosocomial occurrence □Out of hospital:_________________ | | |
| Thrombus site: □upper limb □lower limb □PE □precava □postcava | | |

| Item | Contents | Form of evaluation | Result of evaluation | Notes |
| --- | --- | --- | --- | --- |
| 1 | Whether the nurse completes the VTE risk assessment using the Caprini scale within 24 hours of the patient's ICU admission | On-site observation  View medical records | □YES □NO |  |
| 2 | Whether the nurse assesses the risk for hemorrhage for patients undergoing anticoagulant therapy in each shift | View medical records | □YES □NO |  |
| 3 | Whether the nurse reassesses the risk for hemorrhage and VTE when the patient’s clinical condition changes | On-site inquiry  View medical records | □YES □NO |  |
| 4 | Whether the nurse documents and analyzes the patient's intake and output in each shift to prevent dehydration | View medical records | □YES □NO |  |
| 5 | Whether the nurse performs passive movement of the lower extremities as soon as possible after the patient’s condition becomes stable | View medical records | □YES □NO |  |
| 6 | Whether the nurse assesses if the patient has contraindications to IPC use | On-site inquiry | □YES □NO |  |
| 7 | Whether the nurse can use the IPC accurately | On-site inquiry  View medical records | □YES □NO |  |
| 8 | Whether the nurse assesses the necessity of graduated compression stockings (GCS) every day | On-site inquiry | □YES □NO |  |
| 9 | Whether the nurse monitors if the circumference of stockings fits the lower extremities to avoid folding for patients using compression stockings | On-site inquiry | □YES □NO |  |
